# Perspective of the European Cystic Fibrosis Society on Improving Global Cystic Fibrosis Care

**DOI:** 10.1002/ppul.71720

**Published:** 2026-07-04

**Authors:** Jane C. Davies, Egil Bakkeheim, Audrey Chansard, Pavel Drevinek, Christine Dubois, Jess Matthews, Marta Sheremet, Chris Smith

**Affiliations:** ^1^ National Heart & Lung Institute Imperial College London London UK; ^2^ Department of Paediatrics, Oslo University Hospital National Resource Centre for Cystic Fibrosis Oslo Norway; ^3^ Cystic Fibrosis Europe Brussels Belgium; ^4^ Vaincre La Mucoviscidose Paris France; ^5^ Charles University and Motol and Homolka University Hospital Prague Czechia; ^6^ European Cystic Fibrosis Society Karup Denmark; ^7^ Western Ukrainian Specialised Children's Medical Centre Lviv Ukraine; ^8^ Royal Alexandra Children's Hospital Brighton UK; ^9^ Brighton and Sussex Medical School Brighton UK

**Keywords:** CFTR modulators, cystic fibrosis, education, low and middle income countries, patient registry, standards of care, twinning

## Abstract

**Introduction:**

Outcomes for people with the inherited disease, cystic fibrosis, have improved greatly over the last few decades, but one result of this is a widening gap between regions with high income and well‐resourced healthcare systems and low/middle income countries. The gap stretches from newborn screening programs, provision of standard diagnostics and genetic testing through to access to standard of care therapies.

**Methods and Results:**

This paper describes the various initiatives of the European Cystic Fibrosis Society: our Patient Registry, a Twinning Program linking centers from different regions and our Educational Program.

**Conclusions:**

The European Cystic Fibrosis Society recognizes this as a major issue and seeks through these programs to support colleagues, patients and families in low/middle income countries.

## 1 Introduction

Cystic fibrosis (CF) is now well‐recognized as occurring in all parts of the globe; the over‐stated fact that CF is the “commonest inherited disease of caucasians” perpetuates the myth that the condition does not occur in people from other ancestries and ethnicities. This false belief has contributed to many unrecognized or late‐diagnosed cases with a resultant poorer prognosis [1]. This special edition is timely, building on and helping to further communicate awareness and knowledge of CF as a disease of all regions and ethnic backgrounds. Many low‐ and middle‐income countries (LMICs) face significant challenges in providing effective cystic fibrosis care [2, 3]. These span general, infrastructural challenges [4, 5] and others specifically associated with aspects of the condition and include (i) limited financial resource; (ii) major competing medical and socioeconomic priorities; (iii) fragmented healthcare systems; (iv) geographical and access barriers for people needing to travel to receive care. Specific to CF there may also be: (v) a lack of awareness of clinical presentation coupled with an absence of newborn screening programs; (vi) inadequate diagnostic equipment and lack of access to genetic screening; (vii) high cost of CF medications which in many regions is prohibitive. In this article, we draw on our experience from within the European Cystic Fibrosis Society (ECFS) to consider how we work with the community to improve CF care globally. This article explores how scientific organizations, using ECFS as a case study, can contribute to reducing global disparities in CF care. This narrative review/opinion piece draws on the authors' collective experience as leaders in the ECFS, supplemented by published literature on global CF care disparities and improvement initiatives. We describe initiatives in which ECFS has direct involvement and documented outcomes are described, where available. We go on to identify areas requiring further development.

The ECFS (https://www.ecfs.eu/) is an international community of scientific and clinical professionals committed to improving survival and quality of life for people with CF through promotion of high quality research, education and care. Membership is also open to Patient Organizations, people with lived experience of CF and colleagues from industry. The work of the Society is delivered through a series of major programs including the ECFS Clinical Trials Network (CTN), the European CF Patient Registry (PR), ECFS Standards of Care (SoC), our Education Platform and publication of our Society's clinical/scientific journal, the Journal of *Cystic Fibrosis* (JCF; https://www.cysticfibrosisjournal.com/). Leaders of these programs are co‐opted onto the ECFS Board alongside the President, Vice‐president and a number of elected members. These major programs collaborate and work closely with counterparts in the Cystic Fibrosis Foundation in North America; as examples, CFF's Therapeutic Development Network provides financial support to ECFS CTN and the two trials networks undertake joint, global protocol reviews of trial protocols; several of our procedures have been harmonized in joint SOPs [6]; and our Patient Registries have combined forces on publications [7, 8, 9, 10], some of which have influenced care guidelines.

ECFS supports a number of Working Groups (WG) and Special Interest Groups (SIG); WG currently focus on Newborn Screening, Diagnostics, Exercise, Pulmonary exacerbations, Mental health, Telehealth, Access to New Treatments and advanced cellular models including Airway Epithelia and Intestinal Organoids. SIGs represent CF nurses, pharmacists, dietician/nutritionists, physiotherapists and professionals interested in psychosocial aspects of the condition; communication between SIGs is key, to which end we have recently created an Allied Health ECFS Board position for a rotating representative proposed by SIG leads.

ECFS works closely with Cystic Fibrosis Europe (CFE; https://www.cf-europe.eu/) a federation of national and regional CF Patient Organizations in Europe with members from 39 countries. CFE is a non‐profit organization representing people with CF and their families in Europe, working in close collaboration with other international organizations and being an active partner in several European projects. CFE's mission is to promote and support patient‐centered research, best standards of CF care and to represent and defend the interests of people with CF. ECFS and CFE have joint initiatives including a rolling program of scientific postdoctoral fellowships and the Twinning Program through which CF centers in less well‐resourced areas are supported and which is described in more detail below. Many European countries have well‐established national patient‐representative organizations providing a direct point of contact for people with CF and their families, disseminating knowledge, involving the community in research projects and, in many cases, directly funding research.

## 2 Addressing Challenges Related to Regional Inequities

The socioeconomic and health disparities between European countries are well‐documented (https://health.ec.europa.eu/publications/report-role-healthcare-reducing-inequalities-and-poverty-eu_en). In a multinational cohort of nearly 32,000 individuals with cystic fibrosis, median survival exceeded 50 years, but there was variability and higher national healthcare expenditure was strongly associated with reduced mortality risk after adjustment for key clinical factors [11]. Inequities between European countries are however dwarfed by those which exist globally (https://www.who.int/teams/social-determinants-of-health/equity-and-health/world-report-on-social-determinants-of-health-equity). At times, these can appear insurmountable and lead to “overwhelm inaction,” but there is much that can, indeed should, be done to support people living with CF and our professional colleagues across the globe (Table 1). We discuss below diverse areas with tangible outcomes generated in the field of CF and flag areas requiring further development. We invite ideas and comments from our members and beyond to enrich these activities.

**Table 1:** Ways in which clinical/scientific societies can aid progress globally.

1. Education and knowledge exchange 2. Advocating for diagnostics, appropriate multidisciplinary care and therapies 3. Supporting capacity building in underrepresented regions 4. Supporting research and data sharing eg. through Patient Registries 5. Empowering patients and families 6. Promoting collaboration, inclusion and equal opportunities

### 2.1 Education and Knowledge Exchange

#### 2.1.1 ECFS Conferences

ECFS convenes two international conferences annually: the main annual conference is designed around the multidisciplinary team (MDT) and through invited symposia and oral/poster presentations of submitted abstracts, brings together medical, allied health, scientific and lay members of our community as well as industry, both pharmaceutical and device focussed. Another conference focussing on Basic Science is targeted at early career scientists and is an essential forum to encourage, energize and build collaborative and mentorship relationships. We recognize that travel to, and subsistence at, international conferences is expensive and poses a challenge for many from under‐resourced areas. Selected sessions are therefore recorded and made available through our education platform (detailed below).

#### 2.1.2 The ECFS Education Platform (ECFS‐EP)

Membership of the ECFS, which is substantially discounted for professionals allied to medicine and for members of patient organizations, entitles access to multiple materials through the education platform (https://www.ecfs.eu/education). Unlike travel to conferences, e‐learning resources represent flexible, low cost and time efficient methods of continuing professional development (CPD), education and training so may be specifically of value to those working in low income regions.

The objectives of the education project are to:

Develop a clear *curriculum* appropriate to CF that is designed to enhance education across all disciplines and members of the ECFS (medical care for children and adults, nursing care, physiotherapy, nutrition, psychological care etc.). An abstract describing the multidisciplinary development of the syllabuses [12] was presented at the 2023 ECFS conference and the full publication is currently under journal review.

To assess and understand *educational delivery preferences* a large international survey was conducted over 4 months in 2023 to assess the community's views, experiences and perceptions of e‐learning and the ECFS‐EP [13]. 547 responses were received from 58 countries, many of which were LMICs. Over half of respondents were ECFS members and a wide range of specialties were represented from across CF multidisciplinary teams. Analysis showed that online platforms were used either weekly (34%) or monthly (37%); 54% of respondents had used the ECFS‐EP and the material was rated favorably overall, particularly for content quality. Preferred formats for education were articles and medium length webinars (15–30 min). Data gathered through this survey have played a pivotal role in shaping future activities and priorities to better reflect the needs of the community.

We now seek to respond directly to community‐identified needs and align our educational activities with these priorities through the development of a range of educational materials. One such initiative has been the development of accessible webinar series. Between June 2024 and June 2025, close to 2000 attendance episodes were recorded at ECFS webinars covering a diverse range of topics, highlighting the community's engagement with this mode of educational delivery. Records indicate engagement by WHO income group includes LMICs: India, Jordan, Morocco, Albania, Kosovo, Moldova, Uruguay.

#### 2.1.3 Other ECFS Initiatives

Whilst also advancing knowledge and education, the programs and projects below address most closely Aims 2–4 in Table 1: Advocating for access to diagnostics, multidisciplinary care and therapies; Supporting capacity building; Supporting research and data sharing.

#### 2.1.4 The Twinning Program

The Twinning Project (https://www.ecfs.eu/ecfs-standards-care/twinning), a flagship of the ECFS, is a strategic initiative aimed at enhancing the quality of CF care throughout Europe; as such it is perhaps one of the programs most directly addressing the question posed in this article. It is led by the ECFS in collaboration with CF Europe under the umbrella of the Standards of Care program. The project is based on the 2014 ECFS Standards of Care [14], which provided a comprehensive framework for clinical excellence in specialized CF centers. The Standards also identified significant obstacles to the development of healthcare services in less economically advantaged countries. Multiple surveys focusing on CF care in Eastern Europe have confirmed persistent disparities, contributed to by government under‐prioritization, lack of funding, staff shortages, insufficient staff training, and limited access to modern therapies [15, 16]. The Twinning Project directly addresses some of these systemic weaknesses by pairing experienced CF centers from the ECFS‐CTN (a consortium of 68 high‐performing CF centers) with developing CF sites in need of infrastructure support, clinical collaboration, and mentorship.

The program was launched in 2020 with eight mentor–mentee pairs and rapidly expanded. In 2024, it encompassed 19 additional pairs, reflecting growing interest from both mentor and mentee CF centers. For 2025, the program has been further extended under the umbrella of CF Europe through generous funding from the Cystic Fibrosis Foundation.

The cornerstone of a genuine, long‐term partnership is a deep and reciprocal relationship. Sites are therefore strongly encouraged to arrange mutual visits to build trust, gain an understanding of each other's clinical environments, and align on goals (see Text Box 1). Once the specific needs of each mentee site have been identified, the collaboration expands to include activities tailored to those sites. Mentors and mentees design and participate in hands‐on and virtual training, standardizing clinical protocols, holding online clinical case discussions and developing MDTs, pediatric and adult CF services at the mentee site. Close contacts with patient organizations in both countries further strengthens this international collaboration, ensuring that the patient voice remains central to care improvement strategies. In another crucial initiative, the Twinning program supports the translation and dissemination of ECFS Education Platform e‐learning materials described above. Educational resources have already been translated into the languages of several mentee sites, including Romanian, Ukrainian, Turkish, and Albanian. The process, along with the key insights and learnings derived from it were presented at ECFS 2025, and efforts are ongoing to expand this work through the development of additional language versions [17]. Progress in each partnership is closely monitored through regular reports, focusing on communication between mentors and mentees, bidirectional site visits, training activities, and local MDT improvement. Ultimately, the Twinning Project aims to build a sustainable, collaborative CF care network across Europe, as well as fostering individual pair relationships. By supporting structured, long‐term partnership and integrating local patient advocacy, the Twinning initiative is bridging critical gaps and seeking to ensure that all individuals with CF, regardless of their geographical location, receive the care they deserve.

Text Box 1 **Provided by a clinician from a mentee centre in Ukraine following a team visit to the mentor centre in UK. The centres have been de‐identified.**

*Our cystic fibrosis team had a unique opportunity to visit (mentor) Hospital. This experience proved to be extremely effective and inspiring. During the visit we were able to familiarize ourselves with best practices in multidisciplinary care, patient oriented approaches, and innovative treatment methods that have already demonstrated their effectiveness in improving the lives of people with cystic fibrosis*.

*What was especially valuable for us was the direct communication with the team at the (mentor) Hospital and participation in clinical discussions. We witnessed firsthand how effectively a multidisciplinary team can work and we realized how important daily communication between different specialists is. This became a benchmark for us in building and improving our own team*.

*The practical advice we received on managing complex cases and on organizing long term patient follow up was extremely useful. We have already started to implement some of these approaches in our clinic, which gives us greater confidence in our daily work. The patient oriented approach of the (mentor) team, their attention, openness, and partnership with families made a strong impression on us and motivates us to further develop this model of care in our center*.

*For us this collaboration is not only a great honor but also a powerful incentive to continue improving our work in Ukraine. The experience of the (mentor) Hospital helps us adapt international standards of care to our realities, which will ultimately contribute to better quality of life and long term treatment outcomes for our patients. The visit to the centre gave us the feeling that we are part of a large European community, and that our patients deserve the same level of care as in other countries. Despite the difficult conditions of the war, this experience convinced us that we can continue to develop modern medicine for children with cystic fibrosis and build a future in which they will have the best opportunities for life.*

## 3 The European Patient Registry

The ECFS Patient Registry (ECFSPR; https://pr.ecfs.eu/general-information/) is a common platform for collection of data on CF patients in Europe with the following *purposes*:

a. To measure, survey and compare aspects of cystic fibrosis and its treatment in the participating countries, thereby encouraging new standards of dealing with CF [18, 19].
b. To provide data for epidemiological research [20, 21].
c. To identify special patient groups suitable for multi‐center trials.

The ECFSPR collects data from over 56,000 people with cystic fibrosis (CF) across more than 40 countries in Europe both through direct data entry and submission of national registry datasets (https://pr.ecfs.eu/annual-reports/). Through its ongoing expansion program, the ECFSPR aims to achieve coverage across all 50 WHO‐defined European countries and to develop collaborative projects with cystic fibrosis registries LMICs worldwide. Currently, the ECFSPR includes 47 countries, an increase of 20 since 2014, of which 12 of the new countries are classified as LMICs based on the World Bank definition. This topic is further expanded upon in an accompanying paper in this special edition.

The *impact on patients in LMICs* of collecting data in a registry or contributing to wider collaborations can be significant and transformative in several ways:

1. Improving Standards of Care: By establishing CF registries, LMICs can adopt standardized care protocols, ensuring patients receive consistent and high‐quality treatment and allowing the impacts of change to be monitored.
2. Access to Diagnostic Tools: Through the ECFSPR expansion program, the provision of sweat testing equipment and genetic analysis will enable early and accurate diagnosis of CF, which is crucial for effective treatment and management.
3. Pathway to Novel Therapies: With better diagnostic capabilities and standardized care, patients in LMICs will be better positioned to access new and innovative therapies that are being developed globally.
4. Enhanced Data for Research: Collecting and analyzing data from LMICs will contribute to global CF research, helping to identify unique regional challenges and develop targeted solutions.
5. Support and Resources: The ECFSPR's support in governance, data quality, and reporting will help LMICs build robust healthcare infrastructures, ultimately improving patient outcomes.
6. Public Health Planning: The data collected will aid in public health planning, ensuring that resources are allocated effectively and that healthcare policies are informed by accurate, up‐to‐date information.

In addition to the ECFS‐PR Expansion program, there is focus from within ECFS on Quality Improvement (QI) interventions. Various studies have shown that multifaceted QI initiatives such as education, audit and feedback, and the implementation of protocols and guidelines, can significantly improve CF care in LMICs [3, 22]. These interventions focus on stakeholder engagement, organizational readiness, and adaptability.

## 4 Leveraging Published Standards of Care to Support Advocacy Activities

Colleagues and ECFS members in less well‐resourced countries of Europe and beyond have approached the Society for support seeking to improve the quality of diagnostics and treatment for their patient populations; although difficult to measure, the impact of this support appears to have been of value. Integral in this has been the publication of ECFS Standards of Care documents, which outline, with evidence, the current expectations of CF care provision [23, 24, 25, 26]. Furthermore, in response to an approach co‐ordinated by the advocacy group “Vertex Save Us” (VUS) which was supported by CFE and ECFS, the World Health Organization recently accepted CFTR modulators onto its Essential Medicines list [27] (https://www.who.int/publications/i/item/B09474). It is considered that this will increase high‐level awareness globally and encourage governments/reimbursement agencies to ensure provision of these highly effective medicines; some consider it will influence drug pricing over time [28].

Whilst access to CFTR modulators based on licensed approvals is now supported in almost all European countries, for many LMICs globally, drug costs may place them either out of reach or in conflict with the healthcare requirements of the general population. The manufacturer of the currently licensed modulators, Vertex Pharmaceuticals, runs a donation program to several such countries; ECFS members have requested increased transparency of this program, which is now provided on the company's website (https://www.vrtx.com/medicines/cystic-fibrosis-facts-and-figures/) and ECFS is in discussions around further information for the CF care community. Whilst hugely welcome in some regions, such programs are limited in reach‐ at least currently‐ and regarded by some as being inferior to alternative approaches, such as those outlined in the recent review by Zampoli and colleagues [29]. A less expensive (although, increasing in price) generic of ETI has been available for some time from Argentina; dose‐sparing strategies based on co‐administration of metabolic inhibitors being used in some regions to further decrease costs [30]. An agreement for a second generic at a much lower cost (https://www.londonstockexchange.com/news-article/BXP/affordable-treatment-option-for-cystic-fibrosis/17293901) was announced by members of VUS at the North American CF Conference in Seattle 2025. Rigorous clinical trial data are currently lacking for these generics although a small number of studies have reported in‐use data [30, 31]. ECFS recently convened a new Working Group, “Access to new therapy for all responsive people with cystic fibrosis” (ANT‐WG). Building, in part, on the work of the earlier “Task Force: Speeding up Access to New Therapies” [32] and on the pivotal work of Burgel and colleagues in the French Compassionate Program [33, 34], the new WG brings in new members from under‐resourced regions globally. The ANT‐WG aims to contribute to this area by convening experts around (i) consensus on assessment of responsiveness in people (and/or their cells) with rare variants‐ some of which may respond to CFTR modulators to a lesser extent, (ii) further mapping geographical disparity in access to CFTR modulators, and (iii) leveraging Patient Registries to improve access.

## 5 Conclusion

ECFS is a scientific society, not an organization with easy solutions to ingrained global inequities, nor the sorts of financial resource that would be necessary to address this issue at its root cause. The Lancet Commission [2] called for urgent action to expand access to life‐changing treatments, especially in LMIC and among genetically underrepresented populations. Inherent in this are inclusive clinical trial designs, global regulatory harmonization, the development of coordinated international trial networks and strengthening health systems to deliver comprehensive, equitable CF care including access to medicines. Within Europe access to CFTR modulators equalizes survival between lower and higher income countries [35], thus, optimizing care practices and social determinants of health remains crucial in LMICs. Whilst collaborative partnerships between CF clinical/scientific societies and Patient Organizations have enabled significant progress, addressing persistent global disparities in health outcomes and access to transformative therapies requires the concerted and sustained commitment of governments, healthcare systems, and the pharmaceutical industry. ECFS will strive to continue effective work with multiple partners to play its role in this critical endeavor.

## Author Contributions

**Jane C. Davies:** writing – review and editing, writing – original draft. **Egil Bakkeheim:** writing – review and editing, writing – original draft. **Audrey Chansard:** writing – review and editing, writing – original draft. **Pavel Drevinek:** writing – review and editing, writing – original draft. **Christine Dubois:** writing – review and editing, writing – original draft. **Jess Matthews:** writing – review and editing, writing – original draft. **Marta Sheremet:** writing – original draft, writing – review and editing. **Chris Smith:** writing – review and editing, writing – original draft.

## Conflicts of Interest

The authors declare no conflicts of interest.

## Data Availability

Data sharing not applicable to this article as no datasets were generated or analyzed during the current study.
